# Comparison of haploidentical, matched sibling and matched unrelated donor allo-HCT in adult patients with AML in complete remission with measurable residual disease: a study from the Global committee and the ALWP of the EBMT

**DOI:** 10.1186/s40164-026-00827-8

**Published:** 2026-09-19

**Authors:** Yishan Ye, Jacques-Emmanuel Galimard, Myriam Labopin, Francis Ayuk, Ibrahim Yakoub-Agha, Matthias Stelljes, Urpu Salmenniemi, Thomas Schroeder, Gesine Bug, Maria-Pilar Gallego-Hernanz, Edouard Forcade, Jakob Passweg, Maija Itala-Remes, Simona Piemontese, Jaime Sanz, Ali Bazarbachi, Arnon Nagler, Eolia Brissot, Lin Li, Yi Luo, Jimin Shi, Yanmin Zhao, Mohamad Mohty, He Huang, Fabio Ciceri, Norbert Claude Gorin

**Affiliations:** 1 Bone Marrow Transplantation Center, The First Affiliated Hospital, Zhejiang University School of Medicine, 79 Qingchun Road, Hangzhou, China; 2 EBMT Paris Study Unit, Paris, France; 3 University Hospital Eppendorf, Hamburg, Germany; 4 Centre Hospitalier Universitaire de Lille, Lille, France; 5 University of Münster, Münster, Germany; 6 HUCH Comprehensive Cancer Center, Helsinki, Finland; 7 University Hospital Essen, Essen, Germany; 8 University Hospital Frankfurt - Goethe University, Frankfurt am Main, Germany; 9 Hôpital La Milétrie, Poitiers, France; 10 Centre Hospitalier Universitaire de Bordeaux, Hôpital Haut-Lévêque, Pessac, France; 11 University Hospital Basel, Basel, Switzerland; 12 Turku University Hospital, Turku, Finland; 13 Haematology and BMT, IRCCS Ospedale San Raffaele s.r.l., Milan, Italy; 14 University Hospital La Fe, Valencia, Spain; 15 Department of Medicine, Universitat de València, CIBERONC, Instituto Carlos III, Madrid, Spain; 16 American University of Beirut, Beirut, Lebanon; 17 Chaim Sheba Medical Center, Tel Hashomer, Israel; 18 Service d’Hématologie Clinique et de Thérapie Cellulaire, Sorbonne University, Hôpital Saint Antoine, APHP, 184 rue du Faubourg Saint-Antoine, Paris, 75012 France

**Keywords:** Acute myeloid leukemia, Measurable residual disease, Allogeneic hematopoietic cell transplantation, Donor selection

## To the Editor

Disease relapse is the major challenge for AML patients after allogeneic hematopoietic cell transplantation. Measurable residual disease (MRD) detected by multiparameter flow cytometry (MFC) or molecular biology is the commonly used approach to predict post-transplant relapse in AML. Several studies have reported that the risk of post-transplant relapse significantly increases in patients MRD positive before allo-HCT [1–3].

The choice of the donor is a major issue when planning an allo-HCT. A matched sibling donor (MSD) remains priority, while haploidentical hematopoietic cell transplantation (HAPLO) has surged globally in the recent decade [4]. Recently, an increasing number of studies [5, 6], including ours, which focused on core-binding factor AML transplanted in second complete remission [7] and KMT2A-rearranged AML [8], respectively, have found that HAPLO transplants might exert superior graft-versus-leukemia (GVL) effects than human leukocyte antigen (HLA)-matched donor HCTs especially in high-risk AML. It is therefore of interest to see if HAPLO brings higher benefit to AML patients transplanted in CR but still with positive MRD through better relapse prevention. Little data are available on the key issue of donor selection for AML patients transplanted in MRD + CR [9].

In this international EBMT registry-based multi-center retrospective study, data from 3385 adult AML patients in CR1 or CR2 receiving allo-HCT from 2015 to 2022 in 269 EBMT centers were analyzed (Table S1). 609 HAPLO, 1057 MSD, and 1719 MUD were included. Patients in the HAPLO cohort more frequently had intermediate/high-risk disease than those in the MSD or MUD cohorts according to the European LeukemiaNet (ELN) 2022 risk stratification based on cytogenetic abnormalities (HAPLO, 88.7% vs. MSD, 83.2% vs. MUD, 85.9%). Patients in the HAPLO cohort were more frequently transplanted in CR2 than in either MSD or MUD (HAPLO, 22.2% vs. MSD, 15.2% vs. MUD ,18.8%). In addition, myeloablative conditioning was used less frequently in the HAPLO cohort than in the MSD or MUD cohorts (HAPLO, 43.8% vs. MSD, 55.6% vs. MUD, 50.1%). In this study HAPLOs were restricted to recipients receiving PTCy based GVHD prophylaxis, while PTCy was used in 11.4% of MSD and 8.6% of MUD cohort as the backbone of GVHD prophylaxis, respectively. Notably, the differences in baseline characteristics may represent potential confounders and were adjusted for in multivariable analyses.

**Fig. 1:**
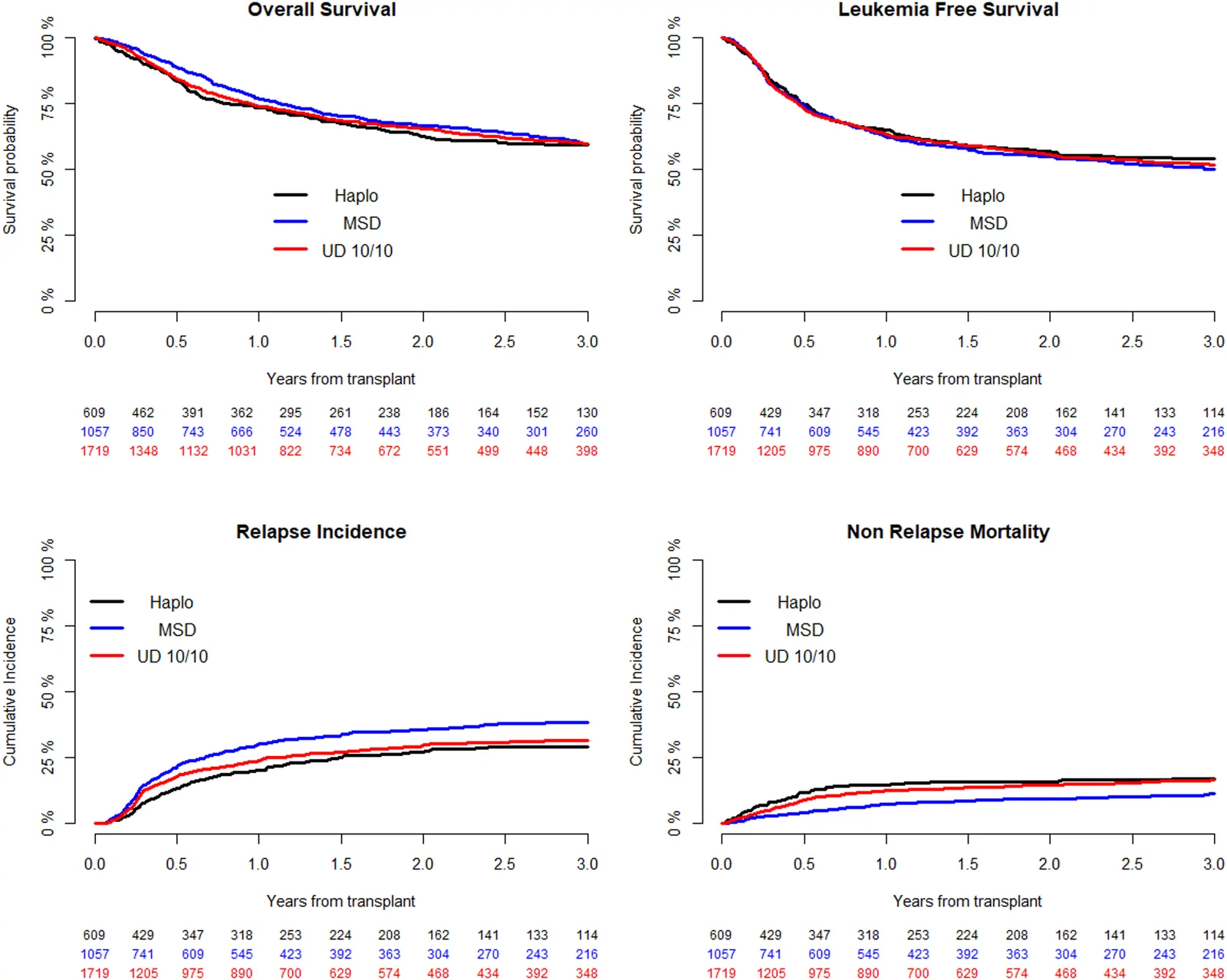
OS, LFS, RI and NRM of the HAPLO, MSD and MUD cohorts

**Fig. 2:**
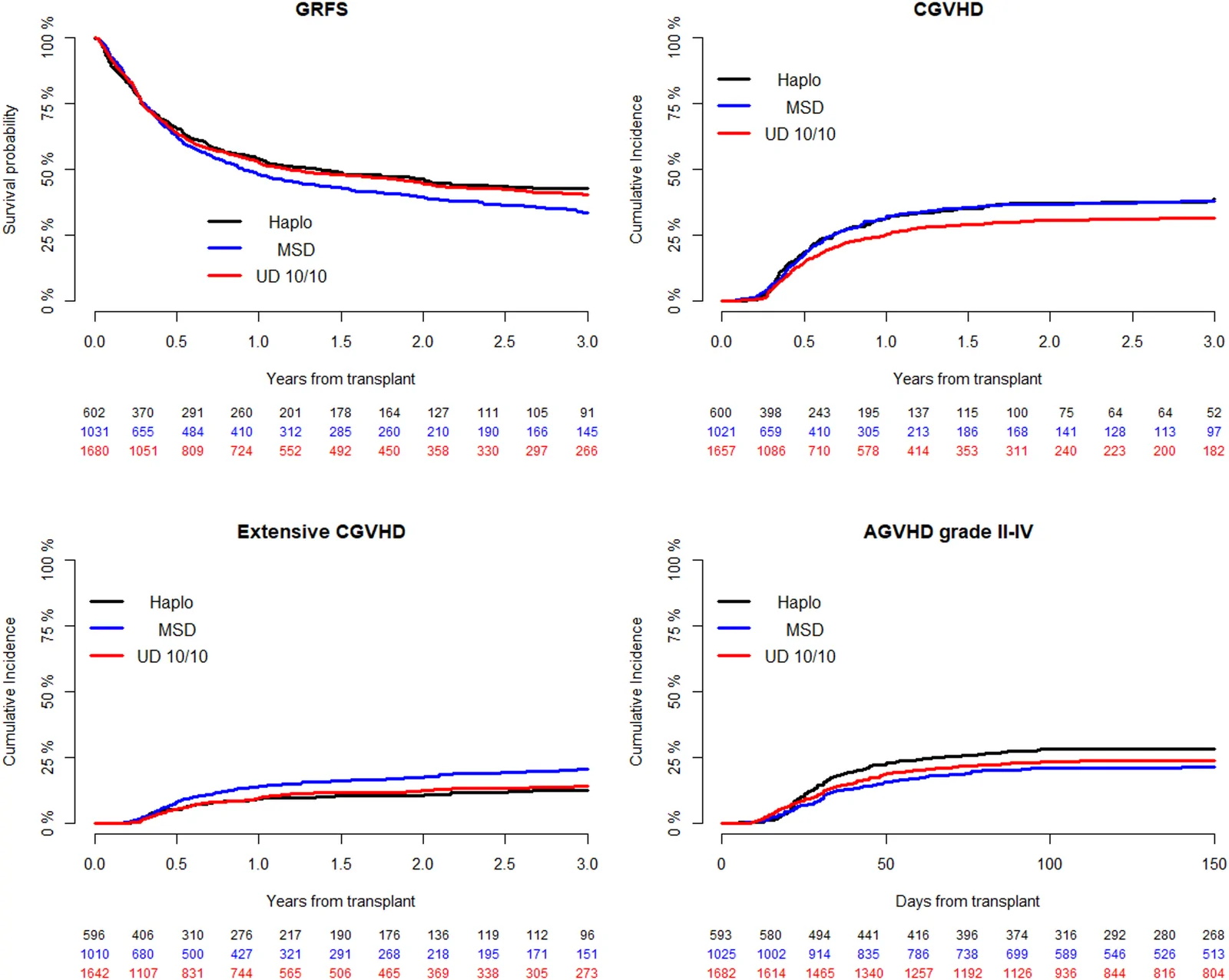
GRFS, cGVHD, extensive cGVHD and grade II-IV aGVHD of the HAPLO, MSD and MUD cohorts

Survival outcomes and transplant complications are shown in Figs. 1 and 2 and data are provided in Supplementary Tables S2 and S3, respectively. The median follow-up is 2.1 years (IQR, 2.1–2.3) for the entire cohort.

On multivariable analysis (Supplementary Table S4) impact of variables on outcomes with competing events were evaluated with a Cox cause-specific model. Compared with HAPLO as reference, both MSD (hazard ratio [HR] = 1.55, 95% confidence interval (95% CI) 1.26–1.91; *p* < 0.01) and MUD (HR = 1.26, 95% CI: 1.03–1.54, *p* = 0.03) were associated with significantly higher RI. Therefore, despite a lower NRM with MSD (HR = 0.63, 95% CI: 0.46–0.84, *p* = 0.004), higher relapse incidence led to inferior LFS compared with HAPLO (HR = 1.19; 95% CI: 1.01–1.41, *p* = 0.048). Concerning GVHD, both MSD (HR = 0.68, 95% CI: 0.55–0.85, *p* < 0.01) and MUD (HR = 0.80 95% CI: 0.66–0.98, *p* = 0.03) were associated with significantly lower incidence of grade II-IV aGVHD compared with HAPLO. Interestingly, although the overall cGVHD incidences were not different between MSD and HAPLO, MSD was associated with significantly higher incidence of extensive cGVHD (HR = 1.77 95% CI: 1.28–2.46, *p* < 0.01) compared with HAPLO. Collectively, MSD was associated with significantly inferior GRFS (HR = 1.22 95% CI: 1.05–1.41, *p* = 0.01) compared with HAPLO. No significant difference was observed between MUD and HAPLO regarding LFS and GRFS. No significant difference was observed among HAPLO, MSD and MUD on OS.

In addition, allo-HCT in CR2 was associated with a significantly higher RI (HR = 1.5 95% CI: 1.27–1.78, *p* < 0.01), lower LFS (HR = 1.21 95% CI: 1.03–1.43, *p* < 0.01), OS (HR = 1.33 95% CI: 1.15–1.54, *p* = 0.02) and GRFS (HR = 1.26 95% CI: 1.11–1.43, *p* < 0.01) compared to allo-HCT in CR1. MAC was significantly associated with a lower RI (HR = 0.75 95% CI: 0.64–0.87, *p* < 0.01), better LFS (HR = 0.78 95% CI: 0.69–0.89, *p* < 0.01), OS (HR = 0.77 95% CI: 0.67–0.90, *p* < 0.01) and GRFS (HR = 0.83 95% CI: 0.74–0.93, *p* < 0.01) compared with RIC. Older patient age (effect for each 10-year increase) was associated with higher NRM (HR = 1.23 95% CI:1.12–1.36, *p* < 0.01) and worse OS (HR = 1.10 95% CI:1.04–1.17, *p* < 0.01). A total of 1069 patients died during the study period (Supplementary Table S5). Relapse/progression of the underlying disease was the most common cause of death in either MSD (71.4%), MUD (59%), or HAPLO (55.7%) cohorts. GVHD was the second most frequent cause of death, which occurred more in the MUD cohort (16.6%) than in either HAPLO (12.9%) or MSD (10.5%) cohorts.

These findings suggest that HAPLO with PTCy may offer superior relapse prevention compared with MSD or MUD for MRD-positive AML patients in remission, and may serve as a good alternative for these patients lacking an MSD. This study has the inherent limitations of its retrospective design. Another important limitation of our study is that PTCy-based GVHD prophylaxis was almost universally used in the HAPLO group but only rarely in the MSD and MUD groups, making PTCy a major confounder that is highly collinear with donor type. Our data cannot fully disentangle whether the observed advantage associated with HAPLO reflects the donor source itself, the use of PTCy, or a combination of both. Dedicated studies directly comparing HAPLO, MSD, and MUD transplants all using PTCy-based GVHD prophylaxis are therefore warranted. In addition, HAPLO was associated with a lower risk of relapse compared with MSD in the current study, but also with higher NRM and similar OS. Thus, the apparent reduction in relapse after HAPLO may at least partly reflect the competing effect of increased NRM, and should therefore be interpreted with caution. Moreover, the lack of detailed information on MRD detection methods in the EBMT registry complicates the interpretation of the results, although even minute levels of MRD positivity have been shown to carry clear prognostic significance [3]. As a well-designed randomized study comparing HAPLO, MSD, and MUD is not yet available, our current registry-based results are of major clinical importance.

## Supplementary Information

Below is the link to the electronic supplementary material.

Supplementary Material 1.

## Data Availability

No datasets were generated or analysed during the current study.
