# Supplementary material for: Comparison of haploidentical, matched sibling and matched unrelated donor allo-HCT in adult patients with AML in complete remission with measurable residual disease: a study from the Global committee and the ALWP of the EBMT
Source: Exp Hematol Oncol. 2026 Sep 19;15:96. doi: 10.1186/s40164-026-00827-8 (PMC13589360; doi:10.1186/s40164-026-00827-8)
Supplement: Supplementary file 1 [file 40164_2026_827_MOESM1_ESM.docx]

**Supplemental data**

**Methods**

**Patients and transplantation procedures**

This was a multi-center analysis using the dataset of the EBMT registry. The EBMT is a voluntary working group of more than 600 transplantation centers. The centers are required to report all consecutive stem cell transplantations and follow-ups once a year. The HCT protocol was approved by the institutional review board of each site, and patients provided informed consent for data collection before transplantation. The study was conducted as per the Declaration of Helsinki and Good Clinical Practice guidelines.

The eligibility criteria for this analysis were as follows: (1) first allogeneic transplant between January 2015 and December 2022; (2) eligible diagnosis: AML patients transplanted in first complete remission (CR1) or second CR (CR2) with positive MRD; (3) allo-HCTs from either MSD, 10/10 MUD (later noted as MUD) or T cell replete HAPLO; (4) HAPLO was restricted to those receiving PTCy based GVHD prophylaxis. The exclusion criteria were allo-HCTs from mismatched UD (<10/10), umbilical cord blood, or grafts with ex-vivo manipulation. The conditioning regimens were defined as myeloablative conditioning (MAC) or reduced-intensity conditioning (RIC) according to the established definitions^8^. Neutrophil and platelet recovery was defined as absolute neutrophil count exceeding 0.5×10^9^/L, and platelet count exceeding 20×10^9^/L for 3 consecutive days without transfusion, respectively. The MRD status was reported to the registry by the participating centers, with no further details provided.

**Endpoints and statistical analysis**

Leukemia-free survival (LFS) was the primary study endpoint. Secondary endpoints were neutrophil and platelet recovery, overall survival (OS), relapse incidence (RI), non-relapse mortality (NRM), grade II-IV and grade III-IV aGVHD, cGVHD and extensive cGVHD, or GVHD-free, relapse-free survival (GRFS). All endpoints were measured from the date of transplantation.

OS was defined as the time from transplant to death from any cause. LFS was defined as survival with no evidence of relapse or progression. GRFS events were defined as the first event among grade III-IV aGVHD, extensive cGVHD, relapse, and death from any cause. Acute and cGVHD were diagnosed and graded using the Revised Glucksberg^9^ and the NIH criteria^10^, respectively. Patients’ characteristics were compared using the Kruskal-Wallis test for continuous variables, and the chi-squared or Fisher’s exact test for categorical variables. The probabilities of OS, LFS, and GRFS were calculated using the Kaplan-Meier estimate. RI, NRM, acute and cGVHD were estimated using cumulative incidence curves. For GVHD, death and relapse were considered competing events. Multivariable analyses were performed using a Cox proportional hazards regression model. In order to adjust the impact of the donor type, we included parameters with unbalanced distribution between the 3 cohorts and those known to potentially influence post-transplant outcomes with the limit of number of variables possible to include. GVHD prophylaxis was not included in Cox regression as related to donor type to avoid collinearity between variables. To take into account the heterogeneity across centers, we introduced a random effect or frailty for each centre into the model.

To avoid possible bias due to the difference in follow-up between the 3 cohorts, all endpoints were censored at 3 years post HCT or at last documented contact. Results were expressed as the hazard ratio (HR) with the 95% confidence interval (95% CI). All tests were two-sided with a type 1 error rate fixed at 0.05. Statistical analyses were performed with R 4.0.2 (R Core Team 2020. R: A language and environment for statistical computing. R Foundation for Statistical Computing, Vienna, Austria. URL <https://www.R-project.org/>).

**Supplementary Table S1 Patient, disease, and transplant characteristics**

|  | **Overall**  **(n=3385)** | **HAPLO PTCy**  **(n=609)** | **MSD**  **(n=1057)** | **MUD 10/10**  **(n=1719)** | **P** |
| --- | --- | --- | --- | --- | --- |
| **Follow-up: years, median [IQR]** | 2.1  [2.1-2.3] | 2.1  [2-2.3] | 2.3  [2.1-2.6] | 2.1  [2-2.3] | Not tested |
| **Patient age: years, median [IQR]** | 55.1  [45.3-63.2] | 55.8  [45.8-63.4] | 52.4  [42.8-60.3] | 57.4  [46.7-64.5] | < 0.001 |
| **Donor age: years, median [IQR]** | 35.5  [27.8-45] | 51.1  [41.6-58] | 28.7  [23.9-36] | 35.5  [27.8-45] | Not tested |
| **Disease status at HCT, n (%)** |  |  |  |  | 0.001 |
| CR1 | 2765 (81.7) | 474 (77.8) | 896 (84.8) | 1395 (81.2) |  |
| CR2 | 620 (18.3) | 135 (22.2) | 161 (15.2) | 324 (18.8) |  |
| **ELN2022 cytogenetic abnormalities, n (%)** |  |  |  |  | 0.04 |
| Favorable | 399 (12.9) | 62 (11.3) | 139 (14.7) | 198 (12.4) |  |
| Intermediate | 2220 (71.7) | 394 (71.6) | 648 (68.5) | 1178 (73.5) |  |
| Adverse | 479 (15.5) | 94 (17.1) | 159 (16.8) | 226 (14.1) |  |
| missing | 287 | 59 | 111 | 117 |  |
| **Type of AML, n (%)** |  |  |  |  | 0.35 |
| De novo | 2917 (86.3) | 529 (87) | 921 (87.3) | 1467 (85.5) |  |
| Secondary AML | 462 (13.7) | 79 (13) | 134 (12.7) | 249 (14.5) |  |
| missing | 6 | 1 | 2 | 3 |  |
| **NPM1, n (%)** |  |  |  |  | 0.99 |
| Absent | 702 (33.1) | 126 (33.2) | 217 (33) | 359 (33.1) |  |
| Present | 1420 (66.9) | 254 (66.8) | 441 (67) | 725 (66.9) |  |
| missing | 1263 | 229 | 399 | 635 |  |
| **FLT3-ITD, n (%)** |  |  |  |  | 0.11 |
| Absent | 1027 (51.5) | 173 (47) | 312 (51.1) | 542 (53.3) |  |
| Present | 968 (48.5) | 195 (53) | 299 (48.9) | 474 (46.7) |  |
| missing | 1390 | 241 | 446 | 703 |  |
| **NPM1/FLT3-ITD, n (%)** |  |  |  |  | 0.7 |
| Absent/Absent | 513 (29.6) | 87 (26.9) | 156 (29.6) | 270 (30.6) |  |
| Absent/Present | 133 (7.7) | 28 (8.6) | 45 (8.5) | 60 (6.8) |  |
| Present/Absent | 423 (24.4) | 76 (23.5) | 129 (24.5) | 218 (24.7) |  |
| Present/Present | 665 (38.4) | 133 (41) | 197 (37.4) | 335 (37.9) |  |
| missing | 1651 | 285 | 530 | 836 |  |
| **Patient sex, n (%)** |  |  |  |  | 0.54 |
| Female | 1602 (47.4) | 276 (45.3) | 503 (47.7) | 823 (47.9) |  |
| Male | 1781 (52.6) | 333 (54.7) | 552 (52.3) | 896 (52.1) |  |
| missing | 2 | 0 | 2 | 0 |  |
| **Female to male, n (%)** |  |  |  |  | <0.001 |
| No | 2847 (84.3) | 504 (82.9) | 797 (75.7) | 1546 (90.1) |  |
| Yes | 529 (15.7) | 104 (17.1) | 256 (24.3) | 169 (9.9) |  |
| missing | 9 | 1 | 4 | 4 |  |
| **Patient CMV, n (%)** |  |  |  |  | 0.54 |
| Female | 1602 (47.4) | 276 (45.3) | 503 (47.7) | 823 (47.9) |  |
| Male | 1781 (52.6) | 333 (54.7) | 552 (52.3) | 896 (52.1) |  |
| missing | 2 | 0 | 2 | 0 |  |
| **CMV donor to recipient, n (%)** |  |  |  |  | <0.001 |
| Negative to Negative | 765 (23) | 101 (17.1) | 203 (19.6) | 461 (27.2) |  |
| Negative to Positive | 715 (21.5) | 138 (23.3) | 160 (15.5) | 417 (24.6) |  |
| Positive to Negative | 291 (8.8) | 30 (5.1) | 105 (10.2) | 156 (9.2) |  |
| Positive to Positive | 1548 (46.6) | 323 (54.6) | 566 (54.7) | 659 (38.9) |  |
| missing | 66 | 17 | 23 | 26 |  |
| **Source of cells, n (%)** |  |  |  |  | Not tested |
| PB | 3083 (91.1) | 476 (78.2) | 959 (90.7) | 1648 (95.9) |  |
| BM | 296 (8.7) | 133 (21.8) | 92 (8.7) | 71 (4.1) |  |
| BM+PB | 6 (0.2) | 0 (0) | 6 (0.6) | 0 (0) |  |
| **Karnofsky score, n (%)** |  |  |  |  | 0.04 |
| < 90 | 874 (26.8) | 136 (23.4) | 263 (25.9) | 475 (28.5) |  |
| >= 90 | 2392 (73.2) | 446 (76.6) | 754 (74.1) | 1192 (71.5) |  |
| missing | 119 | 27 | 40 | 52 |  |
| **Conditioning intensity, n (%)** |  |  |  |  | <0.001 |
| MAC | 1716 (50.7) | 267 (43.8) | 588 (55.6) | 861 (50.1) |  |
| RIC | 1668 (49.3) | 342 (56.2) | 469 (44.4) | 857 (49.9) |  |
| missing | 1 | 0 | 0 | 1 |  |
| **Conditioning regimen, n (%)** |  |  |  |  | Not tested |
| Bu+Other | 35 (1) | 0 (0) | 10 (0.9) | 25 (1.5) |  |
| BuCy based | 314 (9.3) | 3 (0.5) | 161 (15.2) | 150 (8.8) |  |
| BuFlu based | 1191 (35.3) | 85 (14) | 434 (41.1) | 672 (39.3) |  |
| BuFluThio based | 537 (15.9) | 318 (52.3) | 103 (9.7) | 116 (6.8) |  |
| BuMel based | 9 (0.3) | 1 (0.2) | 7 (0.7) | 1 (0.1) |  |
| FluMel based | 313 (9.3) | 38 (6.2) | 87 (8.2) | 188 (11) |  |
| Other | 20 (0.6) | 0 (0) | 9 (0.9) | 11 (0.6) |  |
| TBI based | 429 (12.7) | 91 (15) | 126 (11.9) | 212 (12.4) |  |
| Treo based | 529 (15.7) | 72 (11.8) | 120 (11.4) | 337 (19.7) |  |
| missing | 8 | 1 | 0 | 7 |  |
| **GVHD prevention, n (%)** |  |  |  |  | Not tested |
| CSA based | 292 (8.7) | 9 (1.5) | 147 (14.1) | 136 (7.9) |  |
| CSA+MMF based | 1164 (34.5) | 332 (54.5) | 244 (23.3) | 588 (34.2) |  |
| CSA+MTX based | 1154 (34.2) | 3 (0.5) | 437 (41.8) | 714 (41.6) |  |
| CSA+MTX+MMF based | 25 (0.7) | 9 (1.5) | 4 (0.4) | 12 (0.7) |  |
| MMF+/-TACRO/SIRO based | 515 (15.3) | 235 (38.6) | 116 (11.1) | 164 (9.6) |  |
| MTX+/-TACRO/SIRO based | 68 (2) | 2 (0.3) | 30 (2.9) | 36 (2.1) |  |
| MTX+MMF based | 24 (0.7) | 0 (0) | 6 (0.6) | 18 (1) |  |
| TACRO/SIRO based | 92 (2.7) | 7 (1.1) | 44 (4.2) | 41 (2.4) |  |
| Other | 37 (1.1) | 12 (2) | 17 (1.6) | 8 (0.5) |  |
| missing | 14 | 0 | 12 | 2 |  |
| **In vivo T cell depletion, n (%)** |  |  |  |  | <0.001 |
| No | 1414 (41.9) | 544 (89.3) | 569 (54.4) | 301 (17.5) |  |
| ATG | 1855 (55) | 64 (10.5) | 441 (42.2) | 1350 (78.6) |  |
| Alemtuzumab | 103 (3.1) | 1 (0.2) | 35 (3.3) | 67 (3.9) |  |
| missing | 13 | 0 | 12 | 1 |  |
| **PTCy, n (%)** |  |  |  |  |  |
| No |  |  | 927 (88.6) | 1563 (91.4) | Not tested |
| Yes |  |  | 119 (11.4) | 148 (8.6) |  |
| missing |  |  | 11 | 8 |  |

**Abbreviations:** HAPLO, haploidentical donor; PTCy, post-transplant cyclophosphamide; MSD, matched sibling donor; MUD, matched unrelated donor; IQR, interquartile range; CR, complete remission; ELN, European LeukemiaNet; CMV, cytomegalovirus; PB, peripheral blood; BM, bone marrow; MAC, myeloablative conditioning; RIC, reduced intensity conditioning; Bu, busulfan; Cy, cyclophosphamide; Flu, fludarabine; Thio, thiotepa; Mel, melphalan; TBI, total body irradiation; Treo, Treosulfan; CSA, cyclosporine A; MMF, Mycophenolate mofetil; MTX, methotrexate; TACRO, tacrolimus; SIRO, sirolimus; ATG, Antithymocyte Globulin; PTCy, post-transplant cyclophosphamide;

**Supplementary Table S2. Transplant outcomes of the entire cohort**

| **Transplant outcomes estimation (95% CI)** | | | | | | | | |  |
| --- | --- | --- | --- | --- | --- | --- | --- | --- | --- |
|  | **LFS** | | **OS** | **RI** | | **NRM** | **GRFS** | |  |
| **2 years** | 55.6%  (53.7 – 57.5) | | 65.4%  (63.5 – 67.2) | 31%  (29.3 – 32.8) | | 13.3%  (12.1 – 14.6) | 43.4%  (41.5 – 45.3) | |  |
| **GVHD incidences estimation (95% CI%)** | | | | | | | | | |
| **Acute GVHD (100 days)** | | | | | **Chronic GVHD (2 years)** | | | | |
| Grades II-IV | | Grades III-IV | | | Overall | | | Extensive | |
| 23.7%  (22.2 – 25.1) | | 8.4%  (7.5 – 9.4) | | | 33.9%  (32.1 – 35.7) | | | 13.8%  (12.5 – 15.2) | |

**Abbreviations:** RI, relapse incidence; NRM, non-relapse mortality; OS, overall survival; LFS, leukemia-free survival; GRFS, GVHD-free, relapse-free survival; GVHD, graft-versus-host disease;

**Supplementary Table S3. Transplant outcomes according to donor type**

| **Transplant outcomes estimation (95% CI)** | | | | | | | | | |  |
| --- | --- | --- | --- | --- | --- | --- | --- | --- | --- | --- |
| **Donor type** | **LFS** | | **OS** | | **RI** | | **NRM** | | **GRFS** |  |
| **HAPLO**  **(n=609)** | 56.8%  (52.2 – 61.1) | | 62.7%  (58.1 – 66.9) | | 27.2%  (23.3 – 31.3) | | 15.9%  (12.9 – 19.2) | | 46.4%  (41.9 – 50.9) |  |
| **MSD**  **(n=1057)** | 54.8%  (51.4 – 58.1) | | 66.6%  (63.3 – 69.8) | | 35.6%  (32.4 – 38.8) | | 9.6%  (7.7 – 11.7) | | 39.6%  (36.2 – 42.9) |  |
| **MUD 10/10**  **(n=1719)** | 55.7%  (53 – 58.3) | | 65.6%  (62.9 – 68.1) | | 29.6%  (27.2 – 32) | | 14.7%  (12.9 – 16.6) | | 44.8%  (42.1 – 47.5) |  |
| **GVHD incidence estimation (95% CI)** | | | | | | | | | | |
| **Donor type** | | **Acute GVHD grades II-IV**  **(100 days)** | | **Acute GVHD grades III-IV**  **(100 days)** | | **Chronic GVHD**  **(2 years)** | | **Extensive chronic GVHD**  **(2 years)** | | |
| **HAPLO**  **(n=609)** | | 28.3%  (24.7 – 31.9) | | 10.2%  (7.9 – 12.8) | | 37.3%  (32.9 – 41.7) | | 10.8%  (8.2 – 13.8) | | |
| **MSD**  **(n=1057)** | | 21.2%  (18.7 – 23.8) | | 6.9%  (5.4 – 8.6) | | 36.8%  (33.5 – 40.1) | | 17.6%  (15 – 20.3) | | |
| **MUD 10/10**  **(n=1719)** | | 23.5%  (21.5 – 25.6) | | 8.8%  (7.5 – 10.2) | | 30.8%  (28.3 – 33.4) | | 12.6%  (10.8 – 14.5) | | |

**Abbreviations:** MSD, matched sibling donor; MUD, matched unrelated donor; HAPLO, haploidentical donor; PTCy, post-transplant cyclophosphamide; RI, relapse incidence; NRM, non-relapse mortality; OS, overall survival; LFS, leukemia-free survival; GRFS, GVHD-free, relapse-free survival; GVHD, graft-versus-host disease;

**Supplementary Table S4. Multivariate analysis on factors affecting outcomes for AML patients transplanted with detectable MRD**

|  | **2-year results** | | | | | | | | | |
| --- | --- | --- | --- | --- | --- | --- | --- | --- | --- | --- |
|  | **LFS** | | **OS** | | **RI** | | **NRM** | | **GRFS** | |
|  | **HR**  **(95% CI)** | **P value** | **HR**  **(95% CI)** | **P value** | **HR**  **(95% CI)** | **P value** | **HR**  **(95% CI)** | **P value** | **HR**  **(95% CI)** | **P value** |
| **HAPLO PTCy (ref)** | 1 |  | 1 |  | 1 |  | 1 |  | 1 |  |
| **MSD** | 1.19  (1.01-1.41) | 0.048 | 0.97  (0.80-1.17) | 0.72 | 1.55  (1.26-1.91) | <0.001 | 0.63  (0.46-0.86) | 0.004 | 1.22  (1.05-1.41) | 0.01 |
| **MUD 10/10** | 1.14  (0.97-1.34) | 0.11 | 1.05  (0.88-1.25) | 0.61 | 1.26  (1.03-1.54) | 0.03 | 0.93  (0.71-1.22) | 0.6 | 1.08  (0.93-1.25) | 0.3 |
| **Disease status CR2 vs CR1** | 1.33  (1.15-1.54) | <0.001 | 1.21  (1.03-1.43) | 0.02 | 1.5  (1.27-1.78) | <0.001 | 1.01  (0.77-1.32) | 0.94 | 1.26  (1.11-1.43) | <0.001 |
| **MAC vs RIC** | 0.78  (0.69-0.89) | <0.001 | 0.77  (0.67-0.90) | <0.001 | 0.75  (0.64-0.87) | <0.001 | 0.87  (0.69-1.1) | 0.25 | 0.83  (0.74-0.93) | <0.001 |
| **ELN2022 (cyt)**  **Intermediate** | 1 |  | 1 |  | 1 |  | 1 |  | 1 |  |
| **Adverse** | 1.57  (1.35-1.82) | <0.001 | 1.61  (1.36-1.90) | <0.001 | 1.72  (1.45-2.06) | <0.001 | 1.23  (0.92-1.65) | 0.16 | 1.38  (1.21-1.59) | <0.001 |
| **Favorable** | 0.65  (0.53-0.8) | <0.001 | 0.69  (0.55-0.88) | 0.002 | 0.54  (0.41-0.7) | <0.001 | 0.98  (0.7-1.38) | 0.92 | 0.71  (0.59-0.85) | <0.001 |
| **KPS ≥ 90** | 0.93  (0.81-1.06) | 0.28 | 0.8  (0.69-0.93) | 0.004 | 1  (0.85-1.18) | 0.98 | 0.79  (0.62-0.99) | 0.048 | 0.88  (0.78-0.99) | 0.04 |
| **Patient CMV positive** | 1.14  (1.01-1.29) | 0.04 | 1.20  (1.04-1.38) | 0.01 | 1.16  (1-1.34) | 0.06 | 1.10  (0.88-1.37) | 0.41 | 1.09  (0.98-1.22) | 0.11 |
| **Female to Male** | 0.94  (0.81-1.10) | 0.45 | 1.01  (0.85-1.21) | 0.88 | 0.80  (0.66-0.97) | 0.02 | 1.36  (1.05-1.76) | 0.02 | 1.03  (0.9-1.19) | 0.62 |
| **Age at HCT** | 1.04  (0.99-1.10) | 0.1 | 1.10  (1.04-1.17) | <0.001 | 0.98  (0.92-1.04) | 0.47 | 1.23  (1.12-1.36) | <0.001 | 1.01  (0.96-1.05) | 0.82 |
| **Year at HCT** | 0.97  (0.85-1.12) | 0.71 | 0.98  (0.83-1.16) | 0.84 | 0.95  (0.8-1.12) | 0.54 | 1.02  (0.78-1.32) | 0.89 | 0.95  (0.84-1.07) | 0.38 |

|  | **100-day results** | | | | **2-year results** | | | |
| --- | --- | --- | --- | --- | --- | --- | --- | --- |
|  | **aGVHD II-IV** | | **aGVHD III-IV** | | **cGVHD** | | **Extensive cGVHD** | |
|  | **HR**  **(95% CI)** | **P value** | **HR**  **(95% CI)** | **P value** | **HR**  **(95% CI)** | **P value** | **HR**  **(95% CI)** | **P value** |
| **HAPLO PTCy (ref)** | 1 |  | 1 |  | 1 |  | 1 |  |
| **MSD** | 0.68  (0.55-0.85) | <0.001 | 0.66  (0.46-0.94) | 0.02 | 0.99  (0.81-1.21) | 0.94 | 1.77  (1.28-2.46) | <0.001 |
| **MUD 10/10** | 0.80  (0.66-0.98) | 0.03 | 0.84  (0.61-1.16) | 0.3 | 0.73  (0.60-0.89) | 0.002 | 1.11  (0.80-1.53) | 0.54 |
| **Disease status CR2 vs CR1** | 0.94  (0.76-1.15) | 0.52 | 0.87  (0.62-1.22) | 0.41 | 0.98  (0.80-1.18) | 0.8 | 1.18  (0.89-1.57) | 0.24 |
| **MAC vs RIC** | 1.01  (0.85-1.2) | 0.93 | 0.87  (0.66-1.15) | 0.34 | 1.14  (0.97-1.35) | 0.11 | 0.93  (0.73-1.18) | 0.55 |
| **ELN2022 (cyt)**  **Intermediate** | 1 |  | 1 |  | 1 |  | 1 |  |
| **Adverse** | 1.33  (1.08-1.63) | 0.006 | 1.12  (0.79-1.59) | 0.51 | 1.01  (0.81-1.25) | 0.94 | 1  (0.72-1.39) | 1 |
| **Favorable** | 0.97  (0.76-1.24) | 0.83 | 0.82  (0.53-1.26) | 0.36 | 0.99  (0.79-1.23) | 0.9 | 0.9  (0.65-1.25) | 0.53 |
| **KPS ≥ 90** | 0.9  (0.75-1.08) | 0.25 | 0.67  (0.51-0.88) | 0.004 | 1.11  (0.93-1.32) | 0.27 | 0.89  (0.69-1.15) | 0.39 |
| **Patient CMV positive** | 0.93  (0.79-1.09) | 0.34 | 0.94  (0.73-1.22) | 0.66 | 1.06  (0.91-1.24) | 0.44 | 1.13  (0.90-1.41) | 0.3 |
| **Female to Male** | 0.97  (0.79-1.2) | 0.8 | 1.1  (0.78-1.53) | 0.59 | 1.1  (0.91-1.33) | 0.32 | 1.4  (1.08-1.82) | 0.01 |
| **Age at HCT** | 0.99  (0.93-1.06) | 0.86 | 0.99  (0.89-1.11) | 0.88 | 1.03  (0.97-1.1) | 0.31 | 0.96  (0.88-1.05) | 0.37 |
| **Year at HCT** | 0.83  (0.7-0.99) | 0.04 | 0.8  (0.6-1.06) | 0.12 | 0.85  (0.71-1.01) | 0.07 | 1.02  (0.78-1.34) | 0.89 |

**Abbreviations:** LFS, leukemia-free survival; OS, overall survival; GRFS, RI: cumulative incidence of relapse; NRM, non-relapse mortality; GRFS, GVHD-free, relapse-free survival; GVHD, graft-versus-host disease; aGVHD, acute GVHD; cGVHD, chronic GVHD; HR, hazard ratio; 95% CI, 95% confidence interval; HAPLO, haploidentical donor; PTCy, post-transplant cyclophosphamide; MSD, matched sibling donor; MUD, matched unrelated donor; CR, complete remission; MAC, myeloablative conditioning; RIC, reduced intensity conditioning; ELN, European LeukemiaNet; KPS, Karnofsky score; CMV, cytomegalovirus; HCT, hematopoietic cell transplantation;

**Supplementary Table S5. Causes of death**

|  | **Overall**  **(N = 1069)** | **HAPLO**  **(N = 197)** | **MSD**  **(N = 328)** | **MUD 10/10**  **(N = 544)** |
| --- | --- | --- | --- | --- |
| Relapse | 650 (62.3%) | 108 (55.7%) | 232 (71.4%) | 310 (59%) |
| GVHD | 146 (14%) | 25 (12.9%) | 34 (10.5%) | 87 (16.6%) |
| Infection | 128 (12.3%) | 32 (16.5%) | 32 (9.8%) | 64 (12.2%) |
| VOD | 22 (2.1%) | 8 (4.1%) | 7 (2.2%) | 7 (1.3%) |
| Secondary malignancy | 19 (1.8%) | 2 (1%) | 4 (1.2%) | 13 (2.5%) |
| Cardiac toxicity | 11 (1.1) | 1 (0.5) | 4 (1.2) | 6 (1.1) |
| Pulmonary toxicity | 11 (1.1) | 2 (1) | 2 (0.6) | 7 (1.3) |
| Bleeding | 8 (0.8) | 1 (0.5) | 1 (0.3) | 6 (1.1) |
| Capillary leak syndrome | 1 (0.1) | 1 (0.5) | 0 (0) | 0 (0) |
| CNS toxicity | 7 (0.7) | 1 (0.5) | 2 (0.6) | 4 (0.8) |
| Graft failure | 4 (0.4) | 3 (1.5) | 0 (0) | 1 (0.2) |
| Multi-organ failure | 7 (0.7) | 3 (1.5) | 1 (0.3) | 3 (0.6) |
| Pancreatic toxicity | 1 (0.1) | 0 (0) | 0 (0) | 1 (0.2) |
| Renal toxicity | 5 (0.5) | 1 (0.5) | 1 (0.3) | 3 (0.6) |
| Secondary HLH | 1 (0.1) | 0 (0) | 0 (0) | 1 (0.2) |
| NRM not specified | 23 (2.2) | 6 (3.1) | 5 (1.5) | 12 (2.3) |
| missing | 25 | 3 | 3 | 19 |

**Abbreviations:** HAPLO, haploidentical donor; MSD, matched sibling donor; MUD, matched unrelated donor; GVHD, graft-versus-host disease; VOD, veno-occlusive disease; CNS, central nervous system; HLH, Hemophagocytic Lymphohistiocytosis; NRM, non-relapse mortality
